# Multidrug-resistant *Pseudomonas aeruginosa* aggravates inflammatory responses in murine chronic colitis

**DOI:** 10.1038/s41598-018-25034-2

**Published:** 2018-04-27

**Authors:** Eliane von Klitzing, Ira Ekmekciu, Anja A. Kühl, Stefan Bereswill, Markus M. Heimesaat

**Affiliations:** 1 Department of Microbiology and Infection Immunology, Gastrointestinal Microbiology Research Group, Charité – Universitätsmedizin Berlin, corporate member of Freie Universität Berlin, Humboldt-Universität zu Berlin, and Berlin Institute of Health, Berlin, Germany; 2 Department of Medicine I for Gastroenterology, Infectious Diseases and Rheumatology/Research Center ImmunoSciences (RCIS), Charité – Universitätsmedizin Berlin, corporate member of Freie Universität Berlin, Humboldt-Universität zu Berlin, and Berlin Institute of Health, Berlin, Germany

## Abstract

The World Health Organization has rated multidrug-resistant (MDR) Gram-negative bacteria including *Pseudomonas aeruginosa* (*Psae*) as serious threat to human health. We here addressed whether chronic murine gut inflammation facilitates intestinal MDR *Psae* colonization and whether bacterial infection subsequently worsens colonic immunopathology. Converse to wildtype counterparts, *Psae* colonized the intestines of IL-10^−/−^ mice with chronic colitis following peroral challenge, but did not lead to changes in intestinal microbiota composition. *Psae* infection accelerated both macroscopic (i.e. clinical) and microscopic disease (i.e. colonic epithelial apoptosis), that were accompanied by increased intestinal pro-inflammatory immune responses as indicated by elevated colonic numbers of innate and adaptive immune cell subsets and enhanced secretion of pro-inflammatory cytokines such as TNF and IFN-γ in mesenteric lymph nodes of *Psae*-infected as compared to unchallenged IL-10^−/−^ mice. Remarkably, *Psae*-induced pro-inflammatory immune responses were not restricted to the gut, but could also be observed systemically as indicated by increased TNF and IFN-γ concentrations in sera upon *Psae*-infection. Furthermore, viable commensals originating from the intestinal microbiota translocated to extra-intestinal compartments such as liver, kidney and spleen of *Psae*-infected IL-10^−/−^ mice with chronic colitis only. Hence, peroral MDR *Psae*-infection results in exacerbated colonic as well as systemic pro-inflammatory immune responses during chronic murine colitis.

## Introduction

*Pseudomonas aeruginosa* (*Psae*), constitute opportunistic pathogens, that may cause a variety of nosocomial infections particularly in immunocompromised patients or patients with chronic pulmonary diseases such as chronic obstructive pulmonary disease or cystic fibrosis^1–4^. Patients suffering from ventilator-associated pneumonia or burn wound infections face high mortality rates of over 30%^1^. As Gram-negative bacteria with emerging antimicrobial resistance *Psae* are among several multi-drug resistant (MDR) bacterial species that can be found in a global priority pathogen list issued by the World Health Organization (WHO) in order to help prioritizing the development of novel antimicrobial strategies^5^. Already early studies suggest that besides contaminated respirators and other medical equipment the human gastrointestinal tract might be an important internal source of *Psae* infection in hospitals^6,7^. Furthermore, a recent study revealed that a majority of intensive care unit (ICU) patients with a *Psae* infection displayed prior rectal colonization with the bacterial opportunistic pathogen^8^. Particularly a preceding antimicrobial treatment had been shown to disturb the complex intestinal microbiota composition and therefore to compromise the physiological colonization resistance^9–12^ which, in turn, may enable invading (opportunistic) pathogens including MDR *Psae* to establish within the human gastrointestinal ecosystem^13^. However, valid scientific data concerning the pathogenic potential of *Psae* infection of the intestinal tract are scarce. Our very recent study revealed for the first time that mere intestinal carriage of a clinical MDR *Psae* isolate by otherwise healthy microbiota-depleted wildtype (WT) mice resulted in distinct local as well as systemic pro-inflammatory immune responses^13^. We were further able to demonstrate that with intestinal inflammation associated gut microbiota shifts facilitated murine infection with the enteropathogen *Campylobacter jejuni*^14–17^. Information whether intestinal inflammation may also facilitate stable *Psae* colonization and may even worsen the outcome of the underlying disease are scarce, however. In the present study, we therefore perorally challenged conventionally colonized IL-10^−/−^ suffering from chronic colitis with a clinical MDR *Psae* isolate and assessed intestinal colonization properties of the opportunistic pathogen, shifts in gut microbiota composition, *Psae*-induced intestinal and systemic pro-inflammatory infectious sequelae, and translocation of viable bacteria to extra-intestinal including systemic compartments. In summary, our data indicate that peroral MDR *Psae* infection exacerbates macroscopic and colonic apoptotic sequelae as well as systemic pro-inflammatory immune responses during chronic murine colitis.

## Results

### Chronic colitis facilitates intestinal MDR *P. aeruginosa* infection of mice

**Figure 1:**
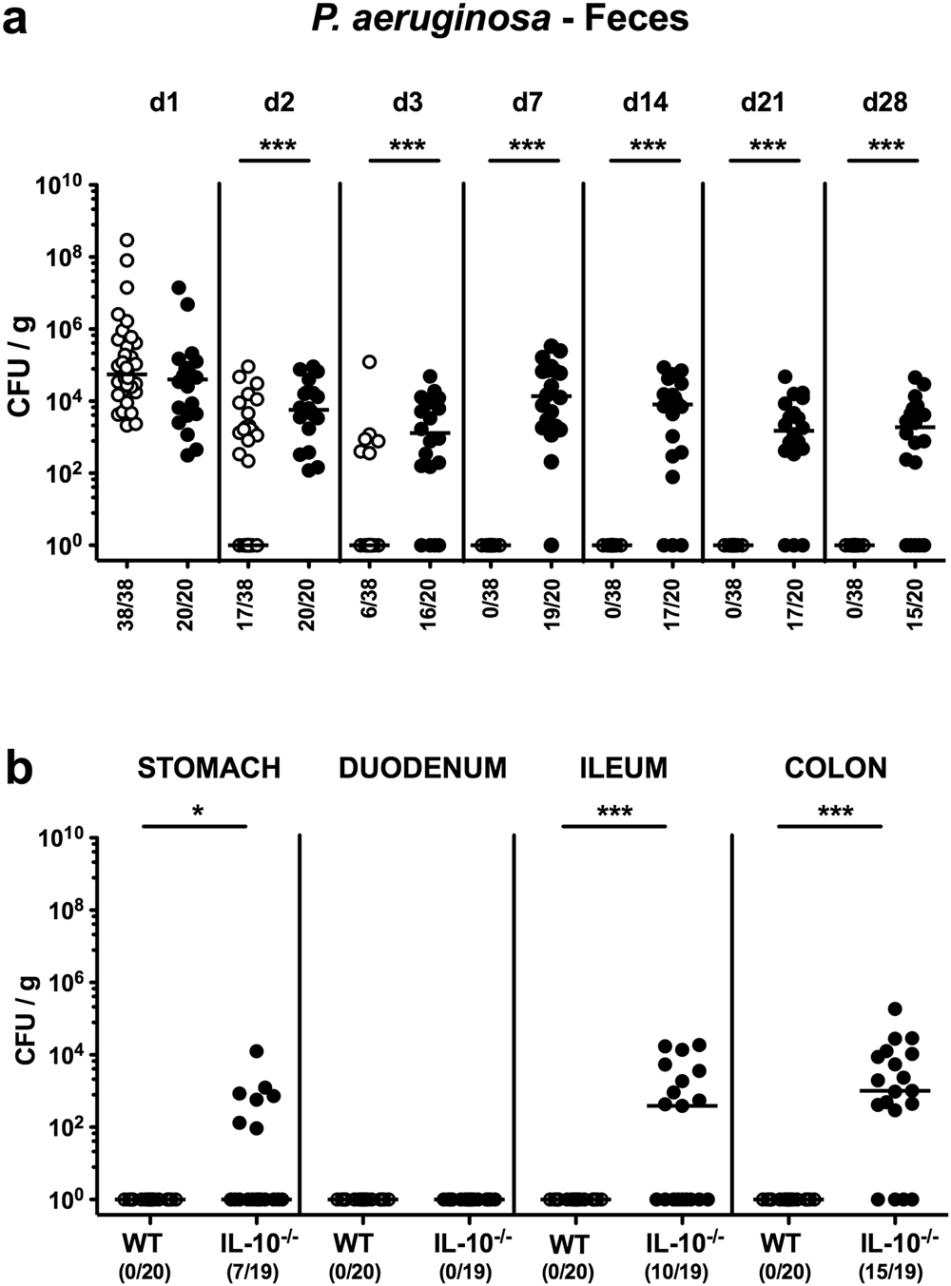
Fecal *P. aeruginosa* loads over time following peroral infection of mice suffering from chronic colitis. Conventionally colonized IL-10^−/−^ mice with chronic colitis (black circles) were perorally infected with a multidrug-resistant *P. aeruginosa* strain on day (d) 0. Wildtype mice without large intestinal inflammation (white circles) served as (infected) controls. (**a**) Intestinal colonization densities were determined in fecal samples until d28 postinfection by culture and expressed as colony forming units per gram (CFU/g). (**b**) Upon necropsy at day 42 postinfection, pathogenic loads were cultured from distinct compartments of the gastrointestinal tract (i.e. stomach, duodenum, ileum and colon). Numbers of mice harboring *P. aeruginosa* out of the total number of analyzed mice, medians (black bars) and significance levels (p-values) determined by Mann Whitney U test are indicated (*p < 0.05; ***p < 0.001). Data shown were pooled from at least three independent experiments.

Conventional IL-10^−/−^ mice with chronic colitis and healthy WT control animals were perorally infected with 10^9^ colony-forming units (CFU) of a clinical MDR *Psae* strain. Whereas WT mice had expelled the pathogen within one week p.i., IL-10^−/−^ mice displayed higher fecal *Psae* loads than their WT counterparts as early as 48 hours p.i. (p < 0.001; Fig. 1a). Even six weeks thereafter, *Psae* could be isolated from the gastrointestinal tract of IL-10^−/−^, but not WT mice (p < 0.05–0.001; Fig. 1b) with most frequent abundances of approximately 79% in the large intestinal tract of IL-10^−/−^ mice at day 42 p.i. (Fig. 1b).

**Figure 2:**
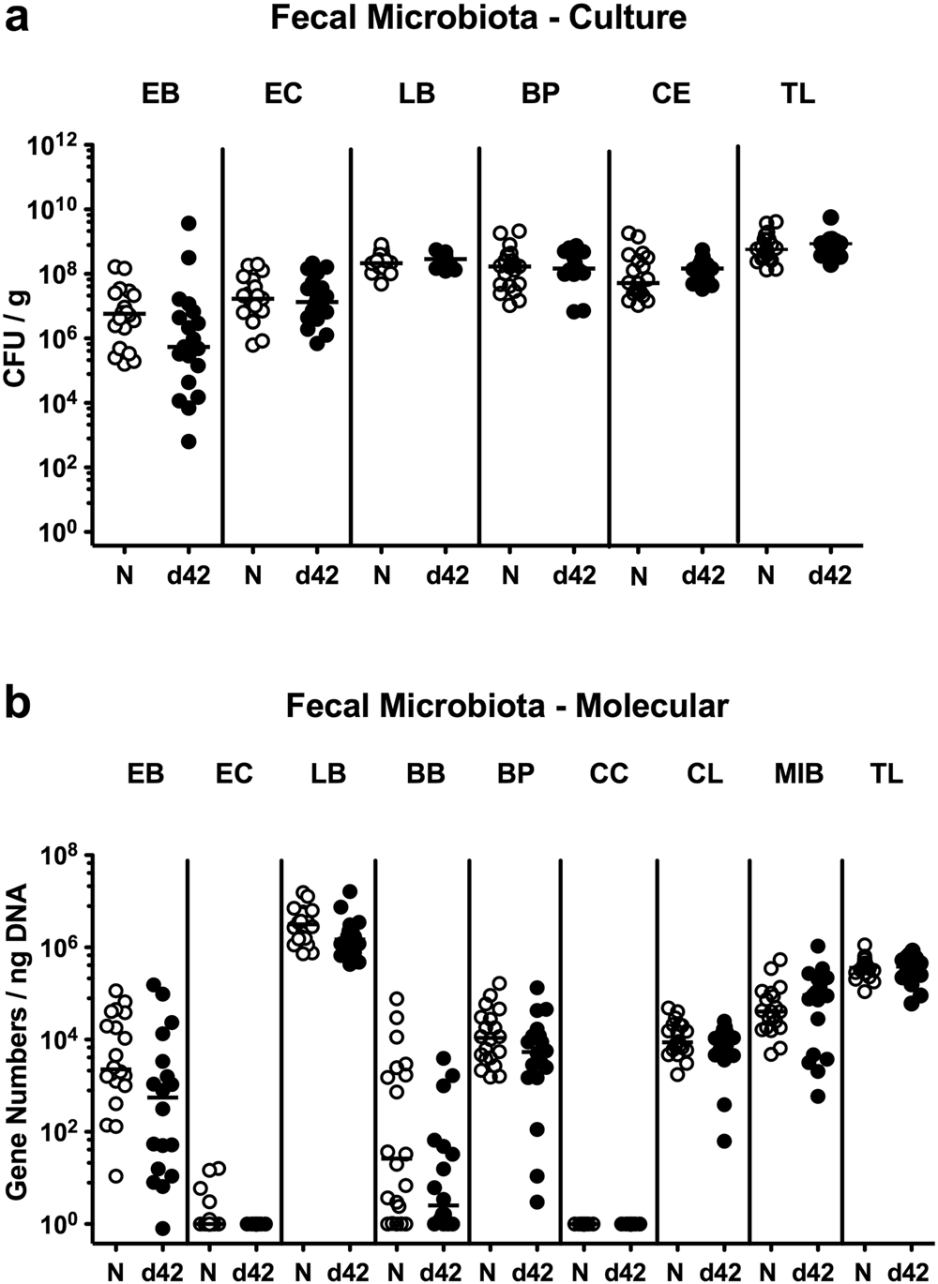
Intestinal microbiota composition following multidrug-resistant *P. aeruginosa* infection of mice suffering from chronic colitis. IL-10^−/−^ mice with chronic colitis (n = 19) were perorally infected with a multidrug-resistant *P. aeruginosa* strain on day (d) 0. Before (N, naive; white circles) and six weeks thereafter (d42 postinfection; black circles) a comprehensive survey of the intestinal microbiota composition was performed on fecal samples applying both (**a**) culture (expressed as colony forming units per gram, CFU/g) and (**b**) 16S rRNA based molecular analyses (expressed as gene numbers per ng DNA) of the total bacterial load (TL) and main intestinal bacterial groups including enterobacteria (EB), enterococci (EC), lactobacilli (LB), *Bacteroides*/*Prevotella* spp. (BP), *Clostridium*/*Eubacterium* spp. (CE), bifidobacteria (BB), *Clostridium coccoides* group, *Clostridium leptum* group (CL) and *Mouse Intestinal Bacteroides* (MIB). Medians are indicated. Data shown were pooled from three independent experiments.

Given potential crosstalk between (opportunistic) pathogens and commensals, we furthermore addressed whether *Psae* association of IL-10^−/−^ mice was accompanied by shifts in intestinal microbiota composition. Both cultural and culture-independent (i.e. molecular 16S rRNA based) analyses of fecal samples revealed that IL-10^−/−^ mice with chronic colitis harbored comparable intestinal loads of the main intestinal bacterial groups before and 42 days after *Psae* challenge (Fig. 2). Hence, chronic IL-10^−/−^ colitis facilitates intestinal infection with MDR *Psae* that does not lead to changes in gut microbiota composition.

### Macroscopic and microscopic sequelae in MDR *P. aeruginosa* infected mice with chronic colitis

We next surveyed macroscopic (i.e. clinical) and microscopic (i.e. histopathological and immunohistochemical) sequelae in *Psae* infected IL-10^−/−^ mice with chronic colitis. Whereas 20% of mice from both the infected and naive cohort of IL-10^−/−^ mice displayed macroscopic and/or microscopic abundances of blood in their feces at day 0 (i.e. immediately before *Psae* infection), overall fecal blood positivity rates were higher in infected as compared to uninfected mice when surveyed until day 42 following bacterial challenge (Supplementary Information). Of note, mean body weights of mice in the *Psae* infected and uninfected cohort did neither differ at day 0 nor at day 42 p.i. (Supplementary Information). Given that intestinal inflammation is accompanied by significant shortening of the inflamed intestine^18^, we measured colonic lengths upon necropsy. A trend towards shorter large intestines could be observed in *Psae* infected as compared to uninfected mice at day 42 p.i.; the difference, however, did not reach statistical significance due to high standard deviations in respective groups (Supplementary Information). We further quantitatively assessed colonic histomorphological sequelae of *Psae* infection in IL-10^−/−^ mice applying a standardized histopathological scoring system^19^. *Psae* infected and uninfected mice with chronic colitis displayed comparable histopathological scores at day 42 p.i., indicative for mild inflammatory cell infiltrates in the mucosa with mild hyperplasia and goblet cell loss (Supplementary Information).

**Figure 3:**
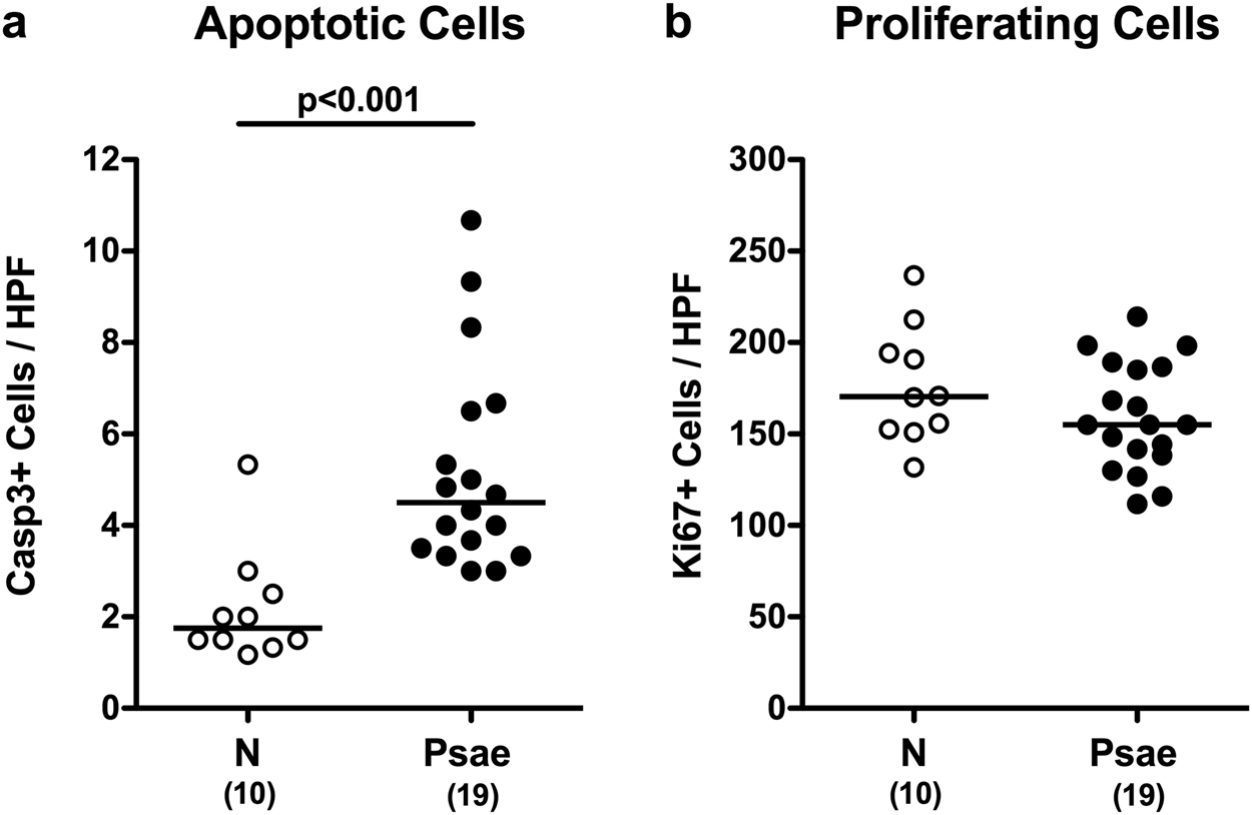
Apoptotic and proliferating colonic epithelial cells in multidrug-resistant *P. aeruginosa* infected mice suffering from chronic colitis. IL-10^−/−^ mice with chronic colitis were perorally infected with a multidrug-resistant *P. aeruginosa* (Psae) strain on day (d) 0. Six weeks thereafter (on d42 postinfection; black circles) the average numbers of colonic epithelial (**a**) apoptotic (positive for caspase 3, Casp3) and (**b**) proliferating cells (positive for Ki67) were determined from six high power fields (HPF, 400x magnification) per animal in immunohistochemically stained large intestinal paraffin sections. Naive (N) IL-10^−/−^ mice served as negative controls (open circles). Numbers of mice (in parentheses), medians (black bars) and significance levels (p-values) determined by the Mann Whitney U test are indicated. Data shown were pooled from three independent experiments.

Given that apoptosis constitutes an established parameter for histopathological evaluation and grading of intestinal inflammation^11^, we next quantitatively assessed apoptotic colonic epithelial cells applying *in situ* immunohistochemistry. Six weeks following *Psae* infection IL-10^−/−^ mice displayed more two times higher apoptotic cell numbers in their large intestines as compared to uninfected counterparts (p < 0.001; Fig. 3a, Supplementary Information). Numbers of Ki67+ colonic epithelial cells, however, did not differ in infected and naive mice (n.s.; Fig. 3b, Supplementary Information), thus indicating comparable proliferative/regenerative properties of the large intestinal epithelia in either murine cohort. Hence, *Psae* infection accelerates both macroscopic (i.e. clinical) and microscopic (i.e. colonic apoptotic) sequelae in IL-10^−/−^ mice suffering from chronic colitis.

### Intestinal immune responses in MDR *P. aeruginosa* infected mice with chronic colitis

**Figure 4:**
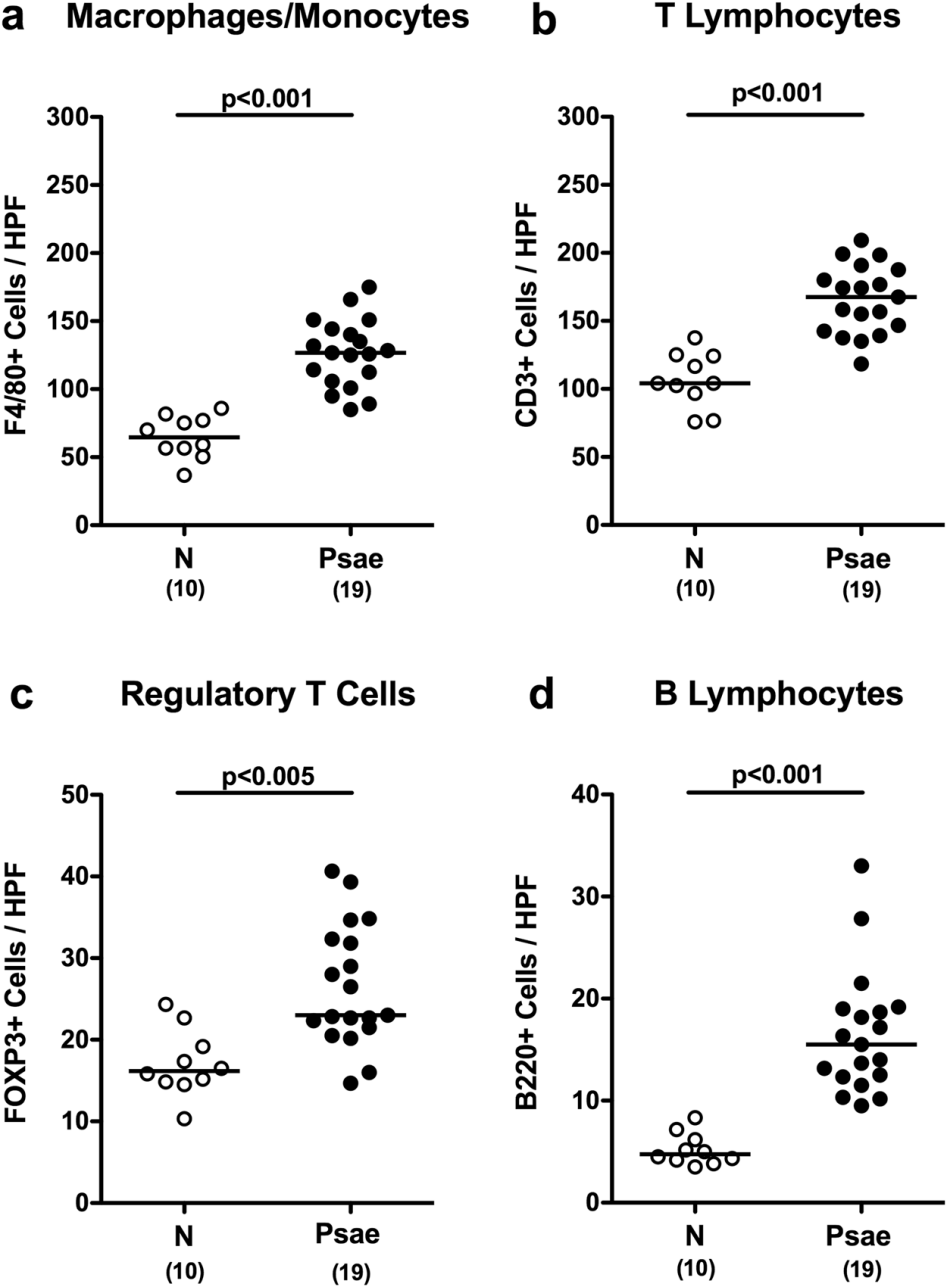
Colonic immune cell responses in multidrug-resistant *P. aeruginosa* infected mice suffering from chronic colitis. IL-10^−/−^ mice with chronic colitis were perorally infected with a multidrug-resistant *P. aeruginosa* (Psae) strain on day (d) 0. Six weeks thereafter (on d42 postinfection; black circles) the average numbers of colonic (**a**) macrophages and monocytes (positive for F4/80), (**b**) T lymphocytes (positive for CD3), (**c**) regulatory T cells (positive for FOXP3), and (**d**) B lymphocytes (positive for B220) were determined from six high power fields (HPF, 400x magnification) per animal in immunohistochemically stained large intestinal paraffin sections. Naive (N) IL-10^−/−^ mice served as negative controls (open circles). Numbers of mice (in parentheses), medians (black bars) and significance levels (p-values) determined by the Mann Whitney U test are indicated. Data shown were pooled from three independent experiments.

**Figure 5:**
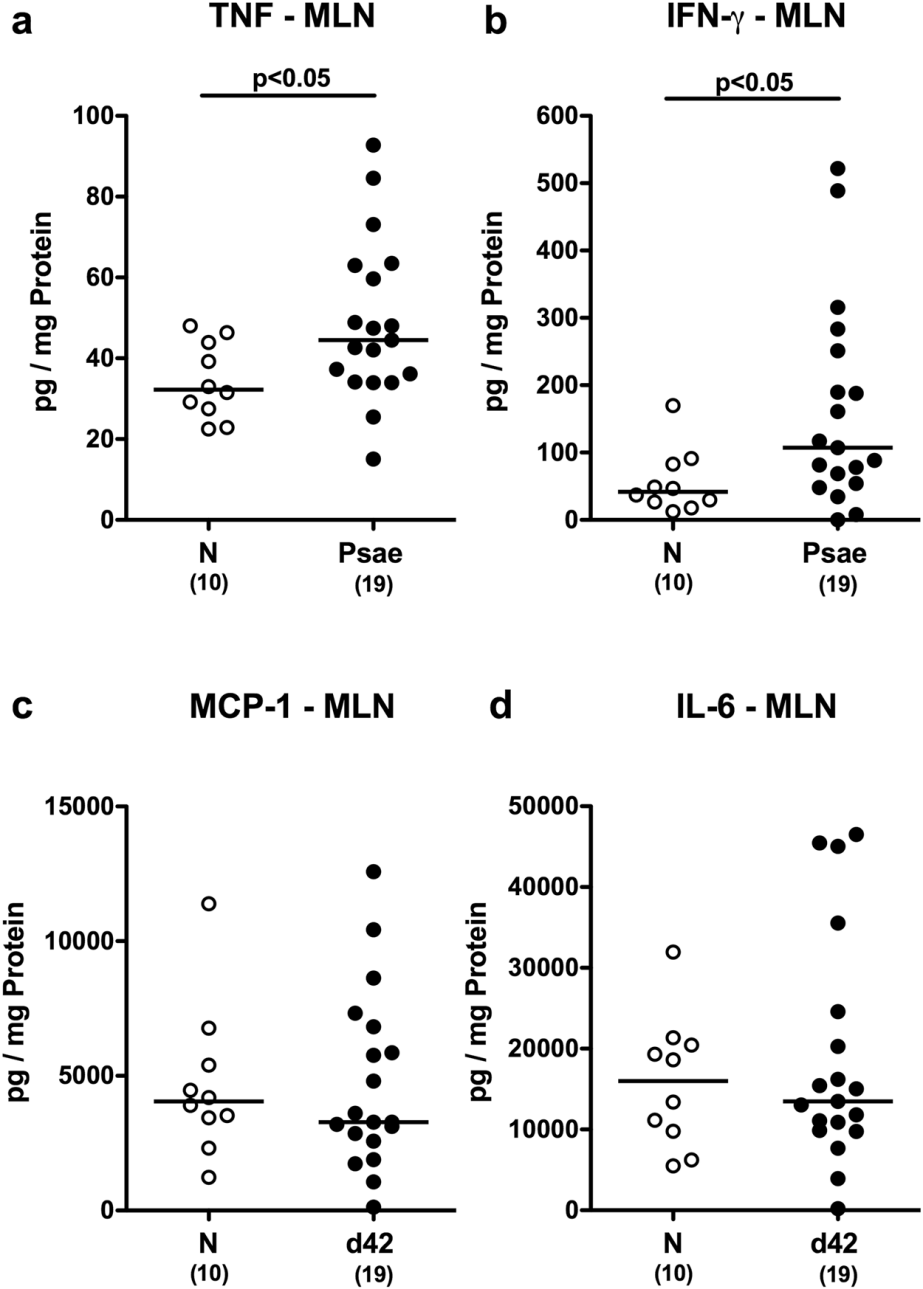
Intestinal cytokine responses in multidrug-resistant *P. aeruginosa* infected mice suffering from chronic colitis. IL-10^−/−^ mice with chronic colitis were perorally infected with a multidrug-resistant *P. aeruginosa* (Psae) strain on day (d) 0. Six weeks thereafter (on d42 postinfection; black circles) pro-inflammatory cytokines including (**a**) TNF, (**b**) IFN-γ, (**c**) MCP-1 and (**d**) IL-6 were measured in supernatants of *ex vivo* biopsies derived from mesenteric lymph nodes (MLN). Naive (N) IL-10^−/−^ mice served as negative controls (open circles). Numbers of mice (in parentheses), medians (black bars) and significance levels (p-values) determined by the Mann Whitney U test are indicated. Data shown were pooled from three independent experiments.

In the following we quantitatively assessed large intestinal immune cell responses upon *Psae* infection of IL-10^−/−^ mice with chronic colitis applying *in situ* immunohistochemistry. Within 42 day p.i., numbers of distinct innate as well as of adaptive immune cell subsets including macrophages and monocytes as well as T lymphocytes, regulatory T cells (Treg) and B lymphocytes, respectively, increased in the large intestinal mucosa and lamina propria of IL-10^−/−^ mice (p < 0.005–0.001; Fig. 4, Supplementary Information). *Psae*-induced increases in large intestinal immune responses were accompanied by elevated secretion of pro-inflammatory cytokines such as TNF and IFN-γ in mesenteric lymph nodes (MLN) of IL-10^−/−^ mice (p < 0.05; Fig. 5a,b), whereas MCP-1 and IL-6 concentration were comparable in *Psae* infected and uninfected mice at day 42 p.i. (Fig. 5c,d). Hence, MDR *Psae* colonization of IL-10^−/−^ mice with chronic colitis is accompanied by increased intestinal pro-inflammatory cytokine responses.

### Systemic immune responses in MDR *P. aeruginosa* infected mice with chronic colitis

**Figure 6:**
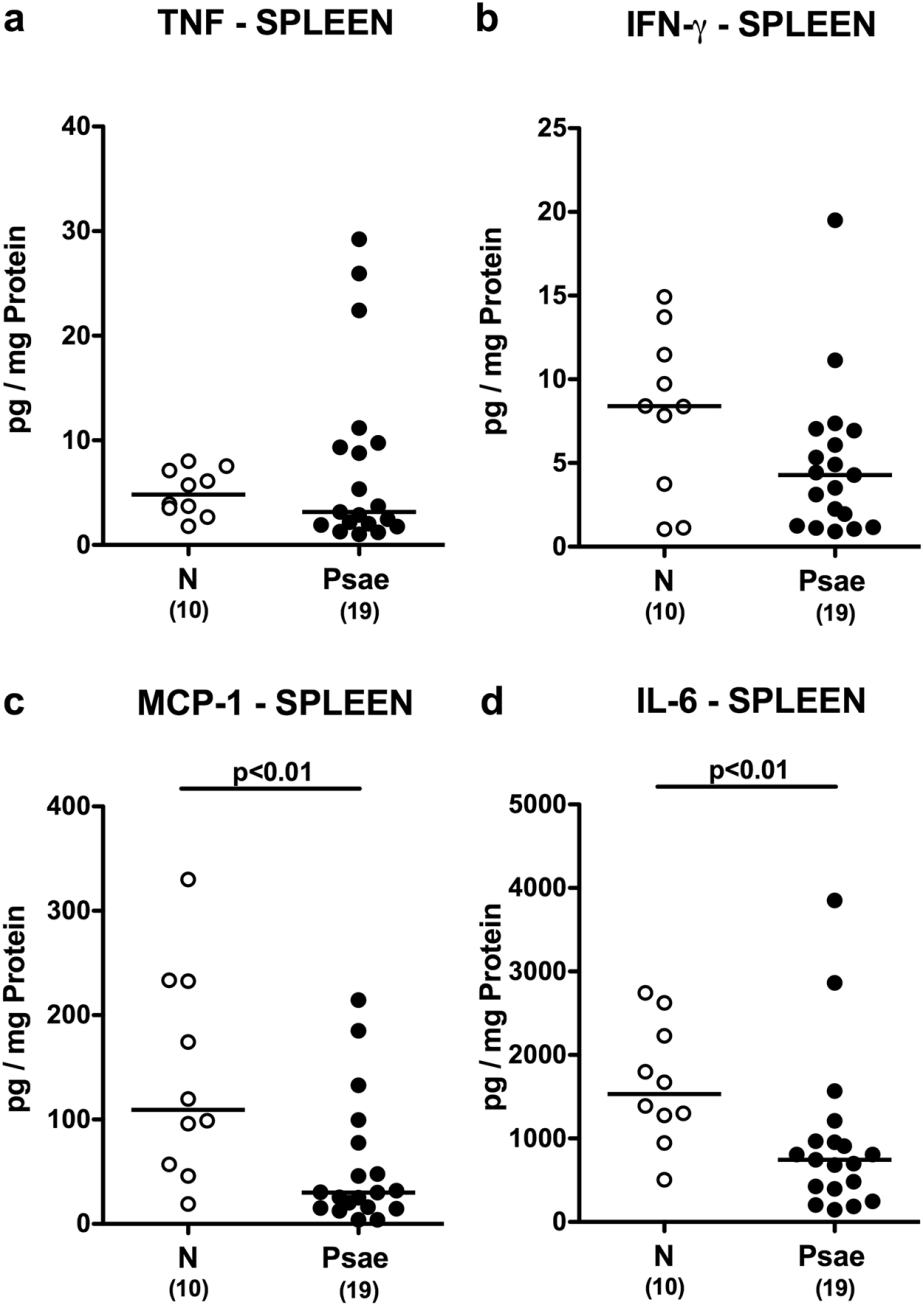
Splenic cytokine responses in multidrug-resistant *P. aeruginosa* infected mice suffering from chronic colitis. IL-10^−/−^ mice with chronic colitis were perorally infected with a multidrug-resistant *P. aeruginosa* (Psae) strain on day (d) 0. Six weeks thereafter (on d42 postinfection; black circles) proinflammatory cytokines including (**a**) TNF, (**b**) IFN-γ, (**c**) MCP-1 and (**d**) IL-6 were determined in supernatants of splenic *ex vivo* biopsies. Naive (N) IL-10^−/−^ mice served as negative controls (open circles). Numbers of mice (in parentheses), medians (black bars) and significance levels (p-values) determined by the Mann Whitney U test are indicated. Data shown were pooled from three independent experiments.

**Figure 7:**
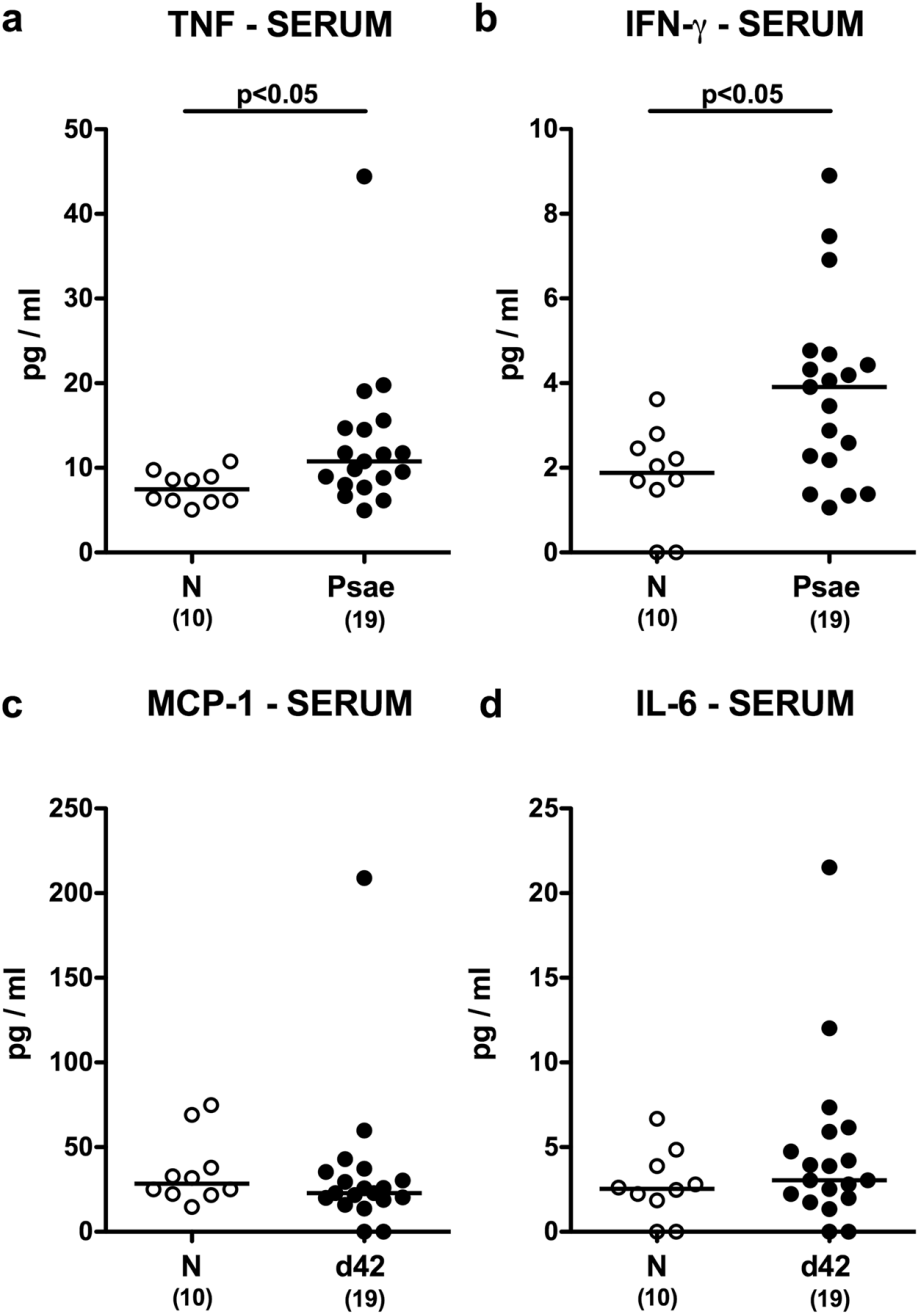
Systemic cytokine responses in multidrug-resistant *P. aeruginosa* infected mice suffering from chronic colitis. IL-10^−/−^ mice with chronic colitis were perorally infected with a multidrug-resistant *P. aeruginosa* (Psae) strain on day (d) 0. Six weeks thereafter (on d42 postinfection; black circles) pro-inflammatory cytokines including (**a**) TNF, (**b**) IFN-γ, (**c**) MCP-1 and (**d**) IL-6 were determined in serum. Naive (N) IL-10^−/−^ mice served as negative controls (open circles). Numbers of mice (in parentheses), medians (black bars) and significance levels (p-values) determined by the Mann Whitney U test are indicated. Data shown were pooled from three independent experiments.

We next assessed whether *Psae*-induced intestinal pro-inflammatory immune responses in mice suffering from chronic colitis could also be observed in systemic compartments such as spleen and serum. Whereas a trend towards lower TNF and IFN-γ levels could be observed in spleens of infected versus uninfected mice with colitis (n.s.; Fig. 6a,b), lower MCP-1 and IL-6 concentrations could be measured in splenic *ex vivo* biopsies at day 42 p.i. as compared to naive IL-10^−/−^ mice (p < 0.01; Fig. 6c,d), Conversely, *Psae* infection resulted in more pronounced secretion of pro-inflammatory cytokines into the circulation as indicated by higher TNF and IFN-γ (p < 0.05; Fig. 7a,b), but comparable MCP-1 and IL-6 concentrations in serum samples derived from infected as compared to uninfected mice with chronic colitis (n.s.; Fig. 7c,d). Hence, MDR *Psae*-induced pro-inflammatory immune responses were not restricted to the intestinal tract, but could also be observed systemically.

### Bacterial translocation in MDR *P. aeruginosa* infected mice with chronic colitis

We finally surveyed whether bacteria originating from the commensal intestinal microbiota had translocated to extra-intestinal and systemic compartments of *Psae* colonized IL-10^−/−^ mice. Whereas no bacterial translocation could be observed in uninfected mice with chronic colitis at all, viable intestinal commensal bacteria such as lactobacilli, enterococci, *E. coli*, *Bacteroides/Prevotella* species and *Clostridium/Eubacterium* species could be cultured from MLN, spleen, liver and kidney in 31.6%, 10.5%, 26.3% and 15.8% of infected IL-10^−/−^ mice, respectively (Supplementary Information). Notably, neither in blood of naive nor *Psae* infected mice with colitis any bacteria could be detected. Hence, viable commensals originating from the intestinal microbiota translocated to extra-intestinal compartments of MDR *Psae* colonized IL-10^−/−^ mice with chronic colitis only.

Taken together, peroral MDR *Psae* infection exacerbates macroscopic and colonic apoptotic sequelae as well as intestinal and systemic pro-inflammatory cytokine responses in IL-10^−/−^ mice with chronic colitis.

## Discussion

The rising increases in incidences of MDR bacterial Gram-negative strains including *Psae* colonizing and infecting humans as well as live stock animals all over the world have resulted in an increased awareness not only of health care professionals, but also of the general public and the political institutions for this global threat of human health and for the actually very limited strategies preventing morbidity and mortality^2,5,20^. It is thus rather surprising, however, that studies addressing the immunopathological consequences of intestinal colonization and infection of vertebrates with *Psae* in health and disease including intestinal inflammation are scare to date.

In an earlier study, ingested *Psae* were detectable in three healthy volunteers up to 6 days thereafter, but without any reported adverse effects^7^. Very recently we were able to show that upon peroral challenge with the same clinical MDR *Psae* isolate used in the present survey, healthy WT mice with a virtually depleted gut microbiota following broad-spectrum antibiotic treatment harbored the opportunistic pathogen at high loads in their gastrointestinal tract, but were clinically uncompromised^13^. This observation is well in line with a previous report by Hentgens *et al*., given that following antibiotic treatment and subsequent *Psae* infection mice were clinically unaffected^9^.

Despite the well-known arsenal of virulence factors and hence pathogenic properties of *Psae*, its potential in initiating and perpetuating pre-existing immunopathological morbidities, particularly in the gastrointestinal tract, is only incompletely understood^13^. Recent surveys revealed that the opportunistic pathogen was more abundant in the gastrointestinal tract of patients suffering from underlying intestinal morbidities such as ulcerative colitis^21^ or irritable bowel syndrome^22^. Remarkably, in our recent study MDR *Psae* induced pronounced pro-inflammatory sequelae in both intestinal and extra-intestinal including systemic compartments in antibiotics-pretreated, but otherwise healthy mice^13^. In addition, we were able to show in another recent survey that acute small intestinal inflammation facilitated MDR *Psae* acquisition by mice harboring a human gut microbiota^23^. The applied inflammation model was, however, too acute to decipher additional inflammatory sequelae of the applied *Psae* strain.

In the present study we further showed that chronic colitis in conventionally colonized IL-10^−/−^ mice (that had not been subjected to antibiotic pretreatment) did not only facilitate murine MDR *Psae* carriage, but also worsened distinct features of the underlying disease as shown on both macroscopic (i.e. clinical) and microscopic levels. In fact, *Psae*-colonized IL-10^−/−^ mice more frequently presented with blood in their feces and exhibited more pronounced colonic apoptosis that were accompanied by higher abundances of innate as well as adaptive immune cell populations in the inflamed colonic mucosa and lamina propria and increased secretion of the pro-inflammatory cytokines TNF and IFN-γ in MLN. Remarkably, accelerated disease was not restricted to the intestinal tract but could also be observed systemically, given that *Psae*-colonized IL-10^−/−^ mice displayed elevated TNF and IFN-γ serum levels. Increased systemic pro-inflammatory cytokines were accompanied by translocation of viable commensal bacteria originating from the intestinal microbiota to extra-intestinal compartments of MDR *Psae*-colonized IL-10^−/−^ mice. This indicates that additional acquisition of the opportunistic pathogen by colitic mice compromised intestinal barrier function subsequently facilitating bacterial spread from the leaky gut. Surprisingly, pro-inflammatory cytokine concentrations were lower in the spleen of *Psae* carrying IL-10^−/−^ mice as compared to non-carriers. This might be explained by increased recruitment of leukocytes from the spleen to the inflamed intestinal tract.

In our present survey, *Psae* could be isolated from the intestines in up to 80% of IL-10^−/−^ mice suffering from chronic colitis, even though mice harbored a conventional gut microbiota that usually protects mice from invading (opportunistic) pathogens^11^. One needs to take into consideration, however, that intestinal inflammatory conditions *per se* might facilitate pathogenic colonization, given that the increased intraluminal abundance of cell debris, for instance, may serve as valuable nutrient sources for specific invading bacteria and may thus provide growth advantages in direct competition with the complex intestinal microbiota for nutrients and niches^16,17,23,24^. Furthermore, both acute and chronic inflammation of the small and large intestines are accompanied by distinct changes in murine microbiota composition providing growth benefits for certain intestinal commensals such as enterobacteria^24–27^, which in turn might change the intraluminal milieu in favor of distinct (opportunistic) pathogenic species. In line, our previous studies revealed that elevated intestinal loads of commensal *Escherichia coli* were sufficient to override physiological colonization resistance and to facilitate murine infection with the enteropathogen *Campylobacter jejuni*^14–16,28^.

Given a potential (bi-directional) crosstalk between (opportunistic) pathogens and intestinal commensals we performed a comprehensive survey of the large intestinal microbiota composition before and after MDR *Psae* challenge applying both culture and culture-independent (molecular) methods. Interestingly, a 6-week intestinal MDR *Psae* carriage by IL-10^−/−^ with chronic colitis did not alter the microbiota within the inflamed colon lumen.

In conclusion, we here show that peroral MDR *Psae* challenge results in intestinal infection that exacerbates chronic colitis as indicated by macroscopic and colonic apoptotic sequelae as well as intestinal and systemic pro-inflammatory cytokine responses in IL-10^−/−^ mice. In ongoing studies we are currently unraveling the distinct intraluminal factors favoring and preventing intestinal MDR *Psae* establishment in health and disease. This may provide novel antibiotics-independent approaches to prevent intestinal colonization with MDR Gram-negative species including *Psae* in vertebrates and to combat subsequent infections of the susceptible host at risk including immunocompromized patients or individuals suffering from intestinal immunopathological morbidities.

## Material and Methods

### Ethical statement

In accordance with the European Guidelines for animal welfare (2010/63/EU), mice were reared, maintained and twice daily monitored assessing clinical conditions and weight loss. The commission for animal experiments headed by the “Landesamt für Gesundheit und Soziales” (LaGeSo, Berlin; registration number G0097/12 and G0039/15) had given approval before start of animal experiments.

### Mice and *P. aeruginosa* infection

Approximately 4 months old conventionally colonized, female IL-10^−/−^ mice with chronic colitis (in C57BL/6j background) and corresponding WT mice were raised in the specific pathogen free unit of the Forschungseinrichtungen für Experimentelle Medizin (FEM, Charité – University Medicine Berlin) and perorally infected with 10^9^ CFU of a MDR *Psae* strain as described earlier^13,23,29^ In brief, the *Psae* isolate had been initially isolated from respiratory fluid of a patient with nosocomial pneumonia and exhibited antimicrobial sensitivity towards colistin and fosfomycin only (provided by Prof. Dr. Bastian Opitz, Charité – University Medicine, Berlin, Germany). The MDR bacterial strain was applied in a total volume of 0.3 mL phosphate buffered saline (PBS; Gibco, life technologies, UK) to mice by oral gavage.

### Cultural analysis of *P. aeruginosa*

Fecal and luminal samples from stomach, duodenum, ileum and colon were plated in serial dilutions onto Columbia agar supplemented with 5% sheep blood (Oxoid, Germany) and Cetrimide agar (Oxoid, Germany) after homogenization in sterile PBS^13,23,29^. Inoculated agar plates were then incubated in an aerobic atmosphere at 37 °C for 48 h to assess *Psae* loads after infection as described earlier^13,23,29^.

### Clinical conditions

On a daily basis, fecal samples derived from each mouse were screened for abundance of fecal blood by the Guajac method (Haemoccult, Beckman Coulter/PCD, Germany) as reported previously^18,29^.

### Sampling procedures

Six weeks following *Psae* infection mice were sacrifized by isoflurane inhalation (Abott, Germany). Cardiac blood and tissue samples from spleen, liver, lung, kidney, MLN, ileum and colon were collected under sterile conditions as described earlier^13,23,29^. After measuring the large intestinal lengths, colonic tissue samples were collected in parallel for histopathological and immunohistochemical analyses as well as for cytokine measurements.

### Histopathology and immunohistochemistry

Histopathological changes observed in 5 µm thin hematoxylin and eosin stained colonic paraffin sections were quantitated applying a standardized histopathological scoring system ranging from 0 to 4 as described by Erben *et al*.^19^. In brief: Score 0: unaffected histomorphology. Score 1: minimal inflammatory cell infiltrates in the mucosa with intact epithelium. Score 2: mild inflammatory cell infiltrates in the mucosa with mild hyperplasia and mild goblet cell loss. Score 3: moderate inflammatory cell infiltrates in mucosa and submucosa with moderate goblet cell loss. Score 4: marked inflammatory cell infiltration into mucosa and submucosa with marked hyperplasia and marked goblet cell loss, multiple crypt abscesses and crypt loss^19^.

To display immune cell response in colonic *ex vivo* biopsies, immunohistochemical staining was performed on five-µm paraffin sections as described earlier^13,23,27,29–32^. In short, primary antibodies against cleaved caspase-3 (Asp175, #9661, Cell Signaling, Netherlands; 1:200) for apoptitic cells, Ki67 (clone 16A8, #652401, BioLegend/Biozol, Germany; 1:200) for proliferating/regenerating cells, F4/80 (clone BM8, #MF48015, Life Technologies, Germany; 1:100) for macrophages/monocytes, CD3 (#IR50361-2, Dako, USA; 1:5) for T lymphocytes, FOXP3 (clone FJK-165, #14–5773, eBioscience, Germany; 1:100) for Tregs and B220 (#14-0452-81, eBioscience; 1:200) for B lymphocytes were used. The average number of positively stained cells within at least six high power fields (HPF, 0.287 mm^2^; 400x magnification) were determined by a blinded independent investigator.

### Cultural survey of intestinal microbiota and bacterial translocation

To survey the composition of the commensal colonic microbiota and the abundance of viable commensals of intestinal origin that had translocated to extra-intestinal including systemic sites, colonic luminal contents and tissue samples derived from MLN, spleen, liver, kidney and lung were homogenized in sterile PBS, streaked out in serial dilutions on agar plates and incubated under aerobic, microaerobic and anaerobic conditions at 37 °C for two to three days as reported earlier^23,24,32,33^. The detection limit of viable bacteria was approximately 100 CFU per g. To assess the appearance of bacteremia, thioglycolate enrichment broths (BD Bioscience, Germany) were inoculated with cardiac blood of individual mice and incubated for seven days at 37 °C and subsequently streaked onto respective agares for further identification as described^23,32^.

### Molecular analysis of the intestinal microbiota

The numbers of 16S rRNA gene copies per ng DNA of main bacterial groups abundant in the murine and human gut microbiota were quantified applying quantitative real-time polymerase chain reaction with species-, genera- or group-specific 16S rRNA gene primers (Tib MolBiol, Germany) as stated elsewhere^11,27,34^. In brief, DNA was extracted from individual fecal samples, quantified by using Quant-iT PicoGreen reagent (Invitrogen, UK) and adjusted to 1 ng per µl^24,35,36^.

### Cytokine detection

Tissue samples of MLN and spleen were transferred to 24-well-flat-bottom culture plates (Falcon, Germany) and incubated in 500 μL serum-free RPMI 1640 medium (Gibco, life technologies) supplemented with penicillin (100 U/mL, Biochrom, Germany) and streptomycin (100 µg/mL; Biochrom) for 18 h at 37 °C. TNF, IFN-γ, MCP-1, and IL-6 concentrations were measured in supernatants and serum samples applying the Mouse Inflammation Cytometric Bead Assay (CBA; BD Bioscience) on a BD FACSCanto II flow cytometer (BD Bioscience) as stated elsewhere^11,23^.

### Statistical analysis

Mean values, medians, and levels of significance were determined using Mann-Whitney-U Test. Two-sided probability (p) values ≤ 0.05 were considered significant. Experiments were repeated at least twice.

## Electronic supplementary material

Supplementary Information
