# Supplementary material for: Multidrug-resistant Pseudomonas aeruginosa aggravates inflammatory responses in murine chronic colitis
Source: Sci Rep. 2018 Apr 27;8:6685. doi: 10.1038/s41598-018-25034-2 (PMC5923287; doi:10.1038/s41598-018-25034-2)
Supplement: Supplementary file 1 — Supplementary Information [file 41598_2018_25034_MOESM1_ESM.pdf]

Multidrug-resistant *Pseudomonas aeruginosa* aggravate intestinal and systemic pro-inflammatory cytokine responses in murine chronic colitis

Eliane von Klitzing, Ira Ekmekciu, Anja A. Kühl, Stefan Bereswill and Markus M. Heimesaat

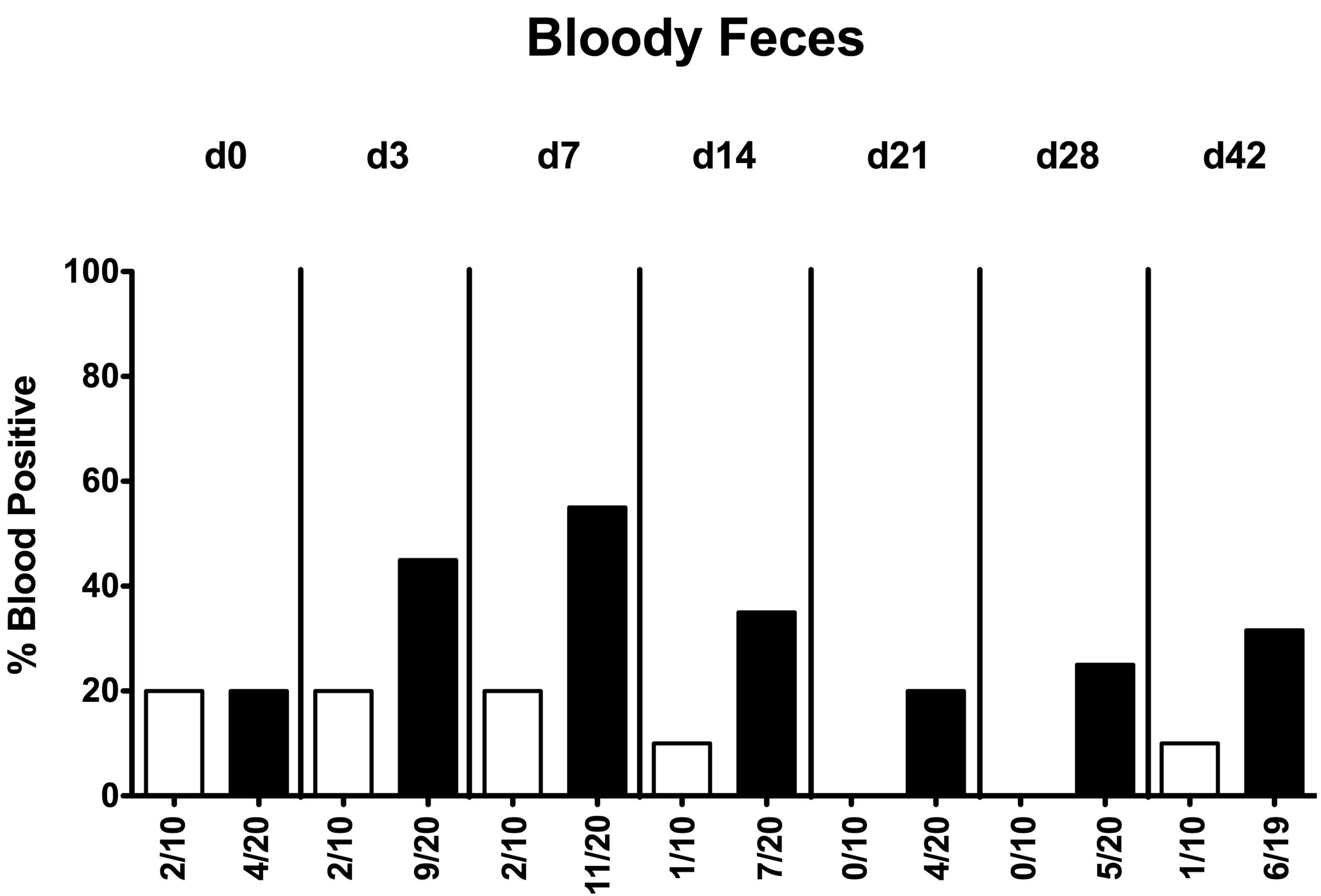

Abundance of blood in fecal samples derived from multidrug resistant *P. aeruginosa* infected mice with chronic colitis. IL-10$^{-/-}$ mice suffering from chronic colitis were perorally infected with a multidrug resistant *P. aeruginosa* strain on day (d) 0 (black bars). Subsequently, the abundance of blood was assessed in fecal samples until necropsy (d42 postinfection) by the Guajac (Haemoccult) method. Naive IL-10$^{-/-}$ mice served as negative (uninfected) controls (white bars). Cumulative blood-positivity rates of three independent experiments (in %) and absolute numbers of mice with bloody feces out of the total number of analyzed animals (on x-axis) are indicated.

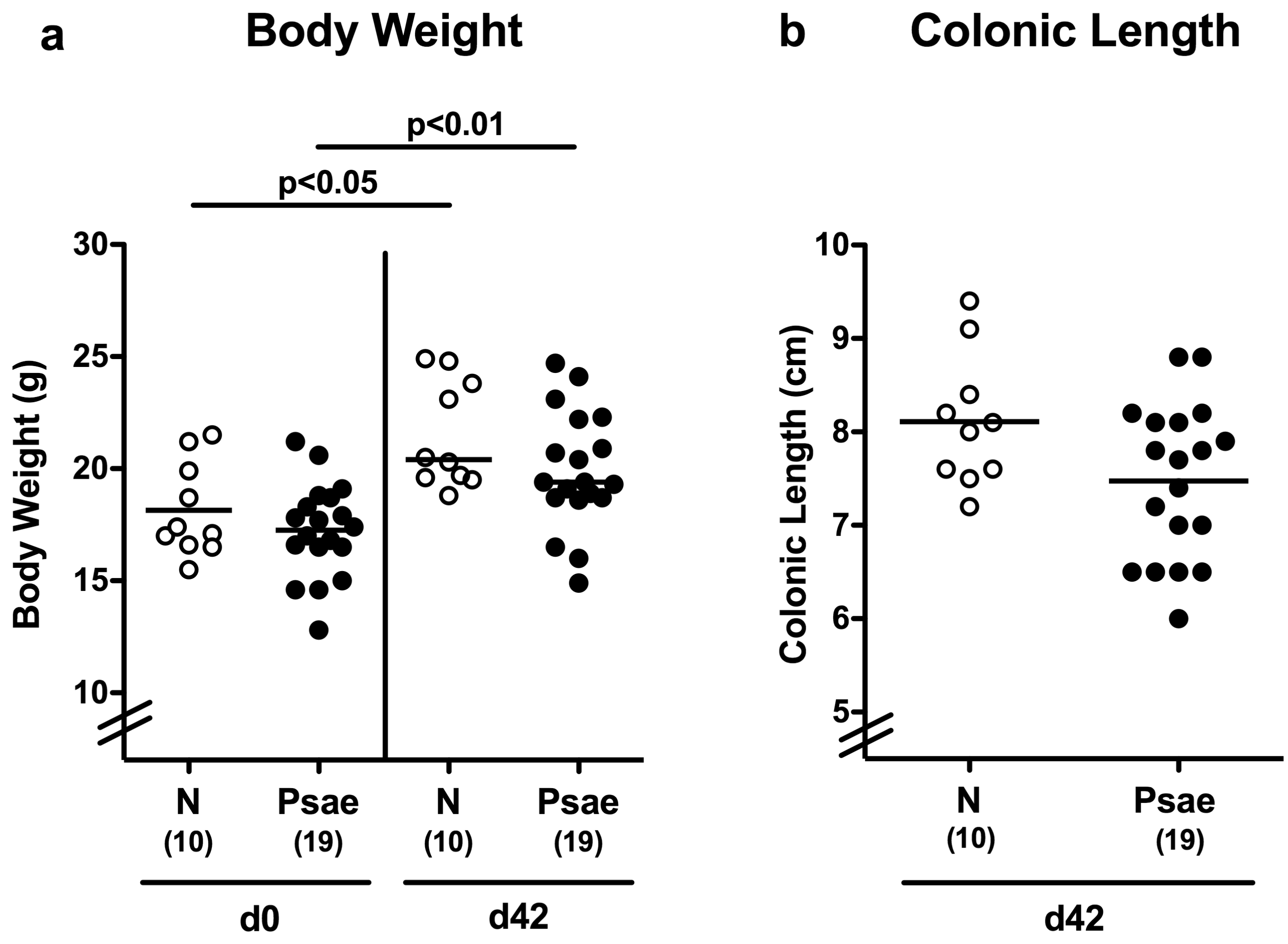

Macroscopic changes in multidrug resistant *P. aeruginosa* infected mice with chronic colitis. IL-$10^{-/-}$ mice suffering from chronic colitis were perorally infected with a multidrug resistant *P. aeruginosa* (Psae) strain on day (d) 0. To assess individual macroscopic changes **(a)** body weights of mice were obtained immediately before (day (d) 0) and six weeks after (d42) Psae infection (black circles) and compared to uninfected, naive (N) controls at respective time points (white circles). Upon necropsy (i.e. at d42 postinfection) colonic lengths (in cm) were measured in Psae infected (black circles) and naive mice (white circles). Numbers of mice (in parentheses), medians (black bars) and significance levels (p-values) determined by the Mann Whitney U test are indicated. Data shown were pooled from three independent experiments.

Histopathology

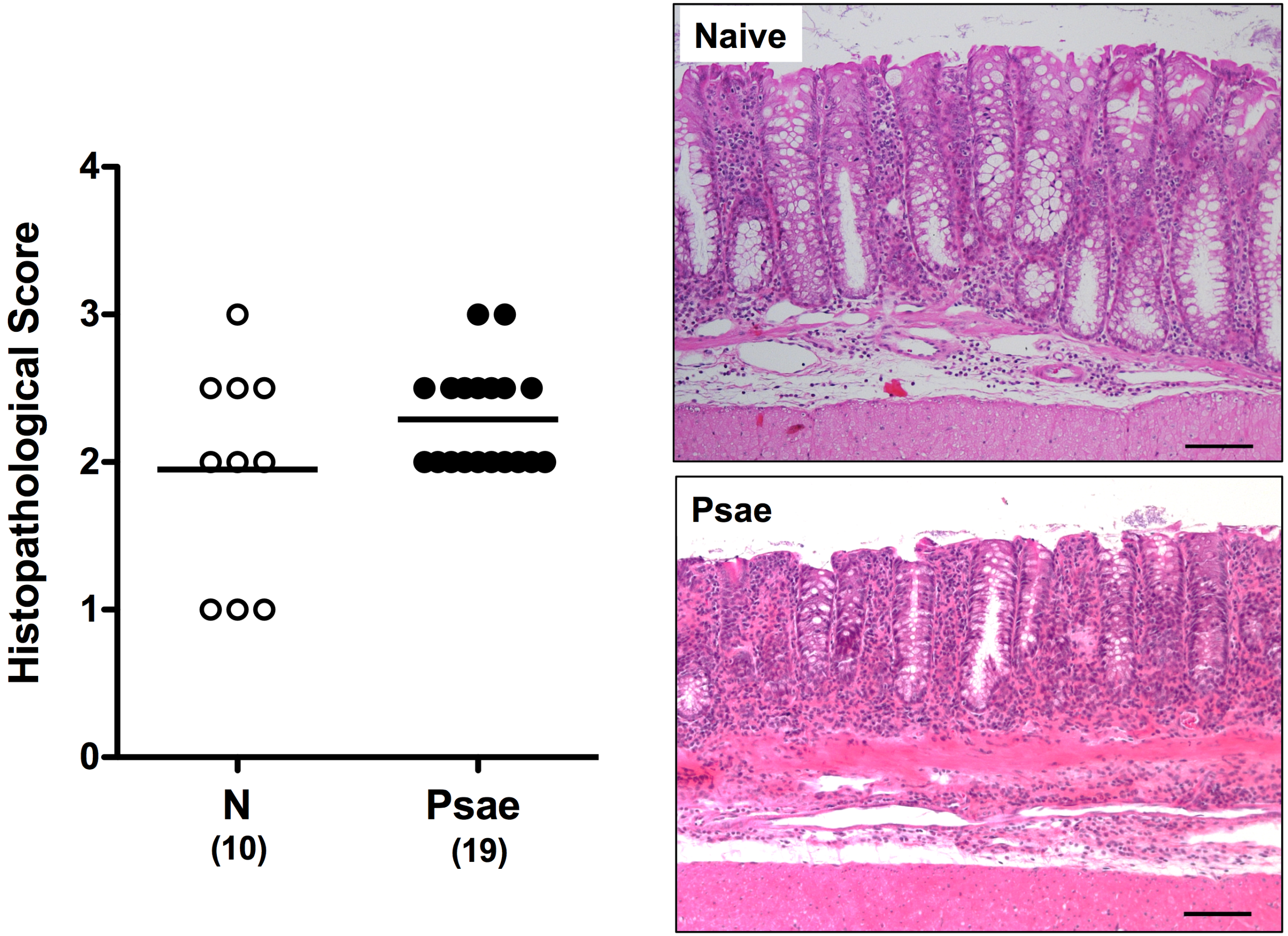

Histopathological changes in large intestines of multidrug resistant *P. aeruginosa* infected mice with chronic colitis. IL-$10^{-/-}$ mice suffering from chronic colitis were perorally infected with a multidrug resistant *P. aeruginosa* (Psae) strain on day 0. Six weeks thereafter histopathological changes were assessed in hematoxylin and eosin stained colonic paraffin sections applying a standardized histopathological scoring system (left). Naive, uninfected mice served as negative controls (N). Numbers of mice (in parentheses) and medians (black bars) are indicated. Data shown were pooled from three independent experiments. Right: Representative photomicrographs illustrate respective histomorphological changes (100x magnification, scale bar 100 μm).

Representative photomicrographs of apoptotic and proliferating epithelial cells, macrophages/monocytes, T lymphocytes, regulatory T cells (Treg) and B lymphocytes in colonic paraffin sections derived from multidrug resistant *P. aeruginosa* infected mice with chronic colitis. IL-10$^{-/-}$ mice suffering from chronic colitis were perorally infected with a multidrug resistant *P. aeruginosa* (Psae) strain on day (d) 0 and sacrifized six weeks thereafter (d42 postinfection; lower panel). Representative photomicrographs illustrate epithelial apoptotic (positive for caspase 3, Casp3) and proliferating cells (positive for Ki67), macrophages and monocytes (positive for F4/80), T lymphocytes (positive for CD3), regulatory T cells (Treg, positive for FOXP3) and B lymphocytes (positive for B220) in immuno-histochemically stained large intestinal paraffin sections (100x magnification, scale bar 100 µm). Naive, uninfected IL-10$^{-/-}$ mice served as negative controls (upper panel).

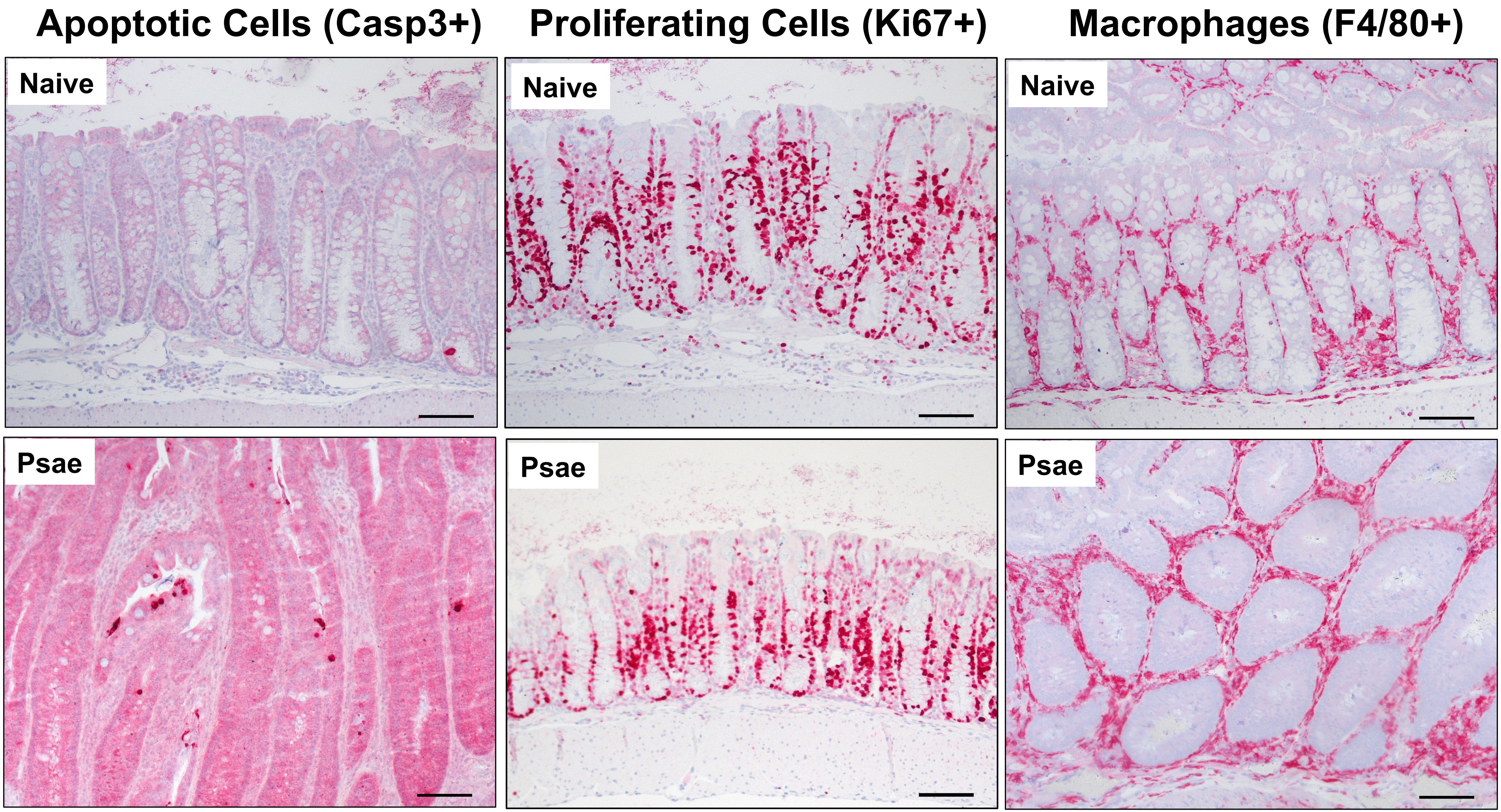
Apoptotic Cells (Casp3+)
Proliferating Cells (Ki67+)
Macrophages (F4/80+)
Naive
Naive
Naive
Psae
Psae
Psae

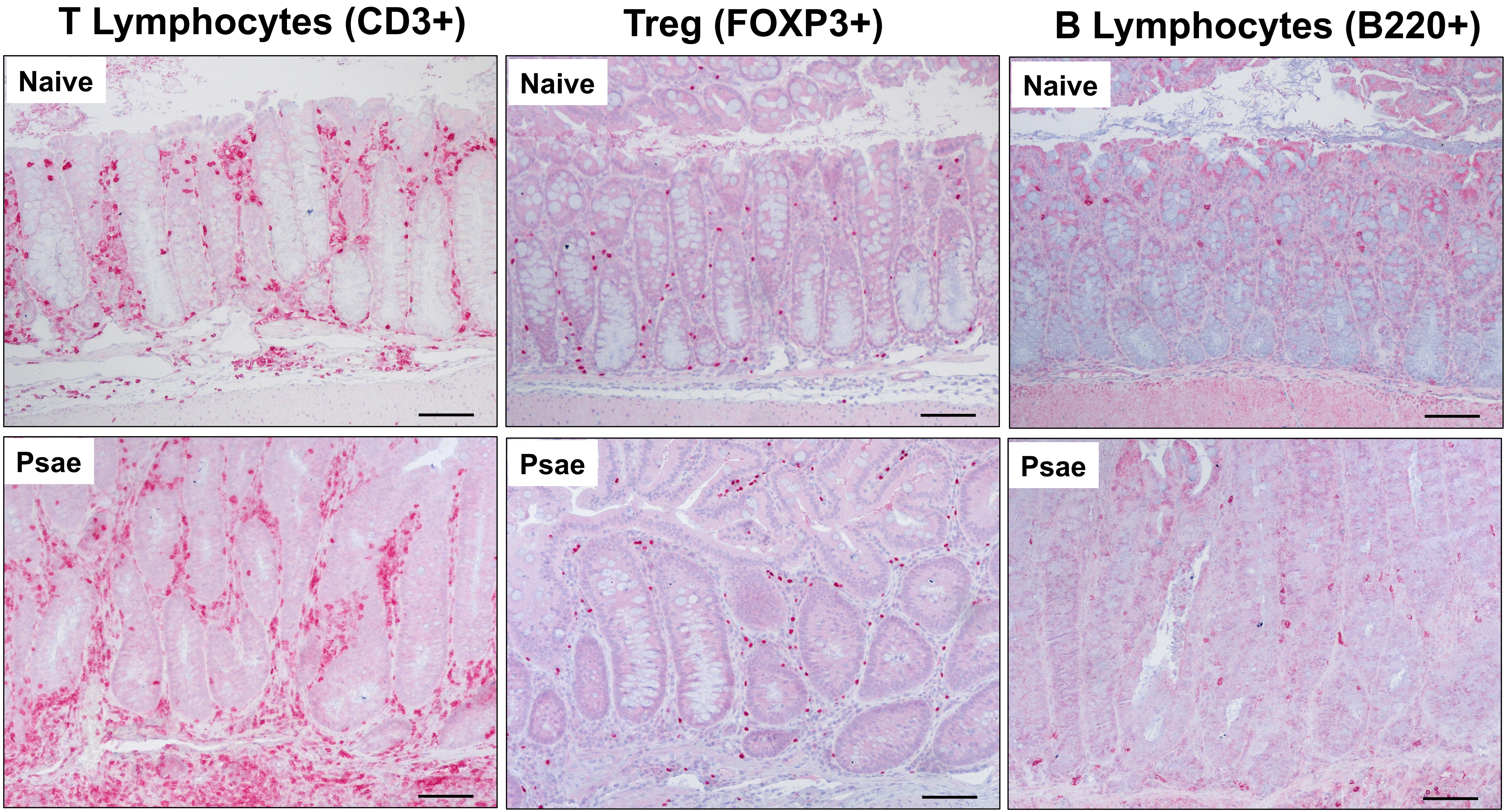

T Lymphocytes (CD3+)
Treg (FOXP3+)
B Lymphocytes (B220+)
Naive
Naive
Naive
Psae
Psae
Psae

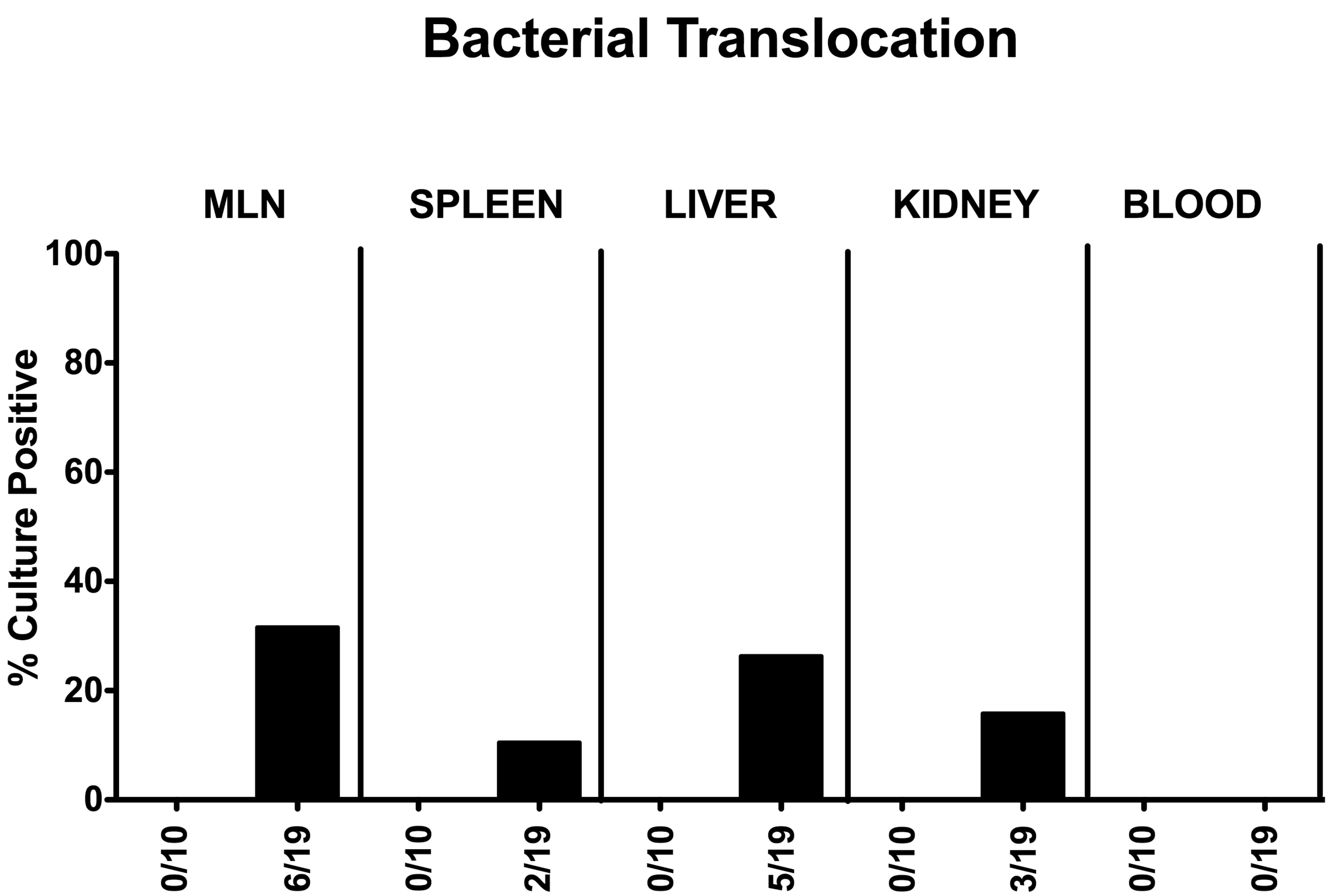

Bacterial translocation in multidrug resistant *P. aeruginosa* infected mice with chronic colitis. IL-$10^{-/-}$ mice suffering from chronic colitis were perorally infected with a multidrug resistant *P. aeruginosa* strain on day (d) 0. Six weeks thereafter (d42 postinfection; black circles) translocation of intestinal bacteria to extra-intestinal and systemic compartments were determined by cultivation of homogenated *ex vivo* biopsies such as MLN, spleen, liver and kidney (direct plating on solid media) as well as of cardiac blood (in thioglycolate enrichment broths) with subsequent subcultivation and species identification. Cumulative relative rates of positive samples (%) out of three independent experiments are indicated.
